# To what extent is antenatal care in public health facilities associated with delivery in public health facilities? Findings from a cross-section of women who had facility deliveries in Nigeria

**DOI:** 10.1186/s12889-023-15688-7

**Published:** 2023-05-04

**Authors:** Benjamin Bukky Ilesanmi, Bola Lukman Solanke, Tosin Olajide Oni, Rasheed Adebayo Yinusa, Omolayo Bukola Oluwatope, Olaoye James Oyeleye

**Affiliations:** 1 Department of Demography and Social Statistics, Obafemi Awolowo University, Ile-Ife, Nigeria; 2 Department of Demography and Social Statistics, Federal University, Birnin-Kebbi, Nigeria; 3 National Centre for Technology Management, Obafemi Awolowo University, Ile-Ife, Nigeria; 4 Hanovia Limited, Abuja, Nigeria; 5 Faculty of Social Sciences, Obafemi Awolowo University, Ile-Ife, Nigeria

**Keywords:** Facility delivery, Private health facilities, Public health facilities, Antenatal care, Delivery care, Maternal health, Nigeria

## Abstract

**Background:**

Facility delivery remains an important public health issue in Nigeria. Studies have confirmed that antenatal care may improve the uptake of facility delivery. However, information is rarely available in Nigeria on the extent to which antenatal care in public health facilities is associated with delivery in public health facilities. The objective of the study was thus to examine the extent of the association between antenatal care in public health facilities and delivery in public health facilities in Nigeria. The study was guided by the Andersen behavioral model of health services use.

**Methods:**

The cross-sectional design was adopted. Data were extracted from the most recent Nigeria Demographic and Health Survey (NDHS). A sample of 9,015 women was analyzed. The outcome variable was the facility for delivery. The main explanatory variable was the antenatal care facility. The predisposing factors were maternal age, age at first birth, parity, exposure to mass media, and, religion. The enabling factors were household wealth, work status, partners’ education, women’s autonomy, health insurance, and, perception of distance to the health facility. The need factors were pregnancy wantedness, the number of antenatal care visits, and the timing of the first antenatal care. Statistical analyses were performed with the aid of Stata version 14. Two binary logistic regression models were fitted.

**Results:**

Findings showed that 69.6% of the women received antenatal care in public health facilities, while 91.6% of them subsequently utilized public health facilities for deliveries. The significant predisposing factors were age at first birth, parity, maternal education, and religion, while household wealth, work status, women’s autonomy, and partners’ education were the significant enabling factors. The timing of the first antenatal contact, pregnancy wantedness, and the number of antenatal care visits were the important need factors.

**Conclusion:**

To a significant extent, antenatal care in public health facilities is associated with deliveries in public health facilities in Nigeria. It is imperative for governments in the country to take more steps to ensure the expanded availability of public health facilities in all parts of the country since their use for antenatal care is well-associated with their use for delivery care.

## Background

Facility delivery refers to childbirth in any medical facility, which may guarantee or fail to guarantee provision of skilled care at birth [1–3]. Notwithstanding, numerous studies have used facility delivery to proxy skilled birth attendance [2, 3]. Facility delivery is important because it avails pregnant women the opportunity of receiving prompt and proper medical care for the treatment of complications such as blood loss during childbirth, obstructed labor, and other complications resulting from pregnancy or childbirth [4, 5]. It also enhances the survival of newborns. This is achieved by reducing the risks of illness and death through prompt detection by skilled birth attendants, of causes of newborn illness, which might result in deaths [6–8], particularly in the first week of birth when most neonatal death occurs [9, 10]. But if the causes of illness are treated rapidly and appropriately, they might not result in child death. In spite of these merits, non-facility deliveries, which actually represent missed opportunities for facility delivery are still dominant in many parts of Africa [4, 11].

Across the world, facility delivery is lowest in Africa and Southern Asia though with substantial variation across the two regions [12]. In Nigeria, the national prevalence of facility delivery is still less than 40% among childbearing women, with a substantial disparity between rural (26%) and urban (61%) areas of the country [13]. It is also noteworthy that there are no positive or negative incentives being implemented in the country to either encourage facility delivery or discourage non-facility delivery. This contrast with the situation in the Philippines, where a ’no home delivery policy’ is being implemented [14]. Facility delivery thus remains an important public health issue in Nigeria. This calls for further research to identify additional practicable strategies for improving the coverage of facility delivery in the country.

One important strategy adopted in Nigeria is to encourage pregnant women to receive antenatal care from skilled personnel either in public or non-public health facilities. This is important because antenatal care is the effective entry point for the utilization of all facility-based maternal and child healthcare services such as facility delivery, postnatal care, and child immunization [15]. Through the health education and counseling provided during antenatal care visits, women who received antenatal care, particularly from skilled providers are provided with vital information on the need to sustain the utilization of other services within the continuum of maternity care. Across developing countries, evidence suggests that these services particularly antenatal and delivery care services are more widely available and utilized in public health facilities compared to private health facilities [16–18]. This may be due to the dominance of public-funded primary healthcare facilities in many developing countries. For instance, in Nigeria, primary healthcare facilities which are under the administration of the local governments constitute more than 80% of health facilities in the country [19]. Though private health facilities could provide inferior or superior care depending on their contexts [20–22], they are usually more expensive and not affordable to many childbearing women, particularly in rural areas [23]. This leaves the majority of pregnant women in developing countries with the option of receiving antenatal and delivery care in public health facilities.

Many existing studies in Nigeria and other countries have established that not all women who receive antenatal care in health facilities subsequently have deliveries in health facilities [24–30]. This has been attributed to several factors such as mistreatment of women during childbirth [31–35], lack of male partner participation, unnecessary regulations imposed by providers [36], and many other health systems challenges such as unavailability of essential drugs and equipment, insufficient number of health personnel, long waiting time at facilities [37], and sub-optimal health management practices [12, 38, 39]. Several other studies have equally provided evidence that antenatal care is indeed crucial to the uptake of facility delivery in developing countries [40–44]. This provides support for the marginal increase in the level of facility delivery in Nigeria from 36% to 2013 to 39% in 2018 bearing in mind that the level of antenatal care from skilled providers also increased from 61 to 67% within the same period in the country [13].

In spite of these numerous studies linking antenatal and delivery care, information is rarely available in Nigeria on the extent to which antenatal care received in public health facilities is associated with delivery in public health facilities. The objective of the study was therefore to examine the extent to which antenatal care in public health facilities is associated with delivery in public health facilities in Nigeria. This was with a view to providing information relevant to strengthening the connections between the different levels of care within the maternity care delivery system in the country. Such information may also inform the women’s health and safe motherhood strategy of the 2021 national policy on population for sustainable development [45], as well as the national health promotion policy which seeks to promote more positive health-seeking behaviors of men and women in the country [46]. The study was guided by the research question: what is the extent of the association between antenatal care in public health facilities and delivery in public health facilities?

The Andersen behavioral model of health services use provides the analytical framework of the study [47, 48]. The framework used in this study depicts facility delivery as dependent on three factors. The first is antenatal care received in public health facilities. This represents an important health behavior that affects the use of facility delivery. The second is the predisposing, enabling, and need factors prescribed by the model. The predisposing factors are the socio-demographic conditions of an individual prior to the need for a particular health service. The enabling factors are the economic, educational, or other resources that facilitate the use of available health services. The need factors are special conditions that create the need for health services. These factors interact within diverse external environments (e.g., place of residence, and geographic region) to influence the utilization of facility delivery. The model was deemed suitable for the study because it incorporates most socio-demographic and health behavior characteristics that may influence facility delivery. Several similar studies have applied the model [49–51] with resultant supportive findings that further justify the wide application of the model.

## Methods

### Design and data

The cross-sectional design was adopted in the study. This entails examining the outcome variable and its covariates on the basis of data collected at the same time from different participants in the most recent Nigeria Demographic and Health Survey (NDHS). The survey was conducted in 2018 as the seventh round of the Demographic and Health Survey (DHS) program in Nigeria. Until 2023 when the eighth round is expected to be conducted, the 2018 NDHS remains the most recent and provides valid estimates of basic demographic and health characteristics in the country. The step-by-step methods employed in conducting the survey have been widely published and could be retrieved online via https://dhsprogram.com/pubs/pdf/FR359/FR359.pdf. The 2018 NDHS consists of diverse datasets such as women, men, kids, and household recode files. The dataset utilized in this study was the individual recode file. This contained individual women’s data. This covered 41,821 women of childbearing age [13]. However, three groups of women were excluded from the study. These are women who had no child delivery in the last five years preceding the survey (20,029), women who had non-facility deliveries (12,700), and those who gave birth in a facility owned by a non-government organization (91). This study thus included 9,015 childbearing women who had facility delivery during their most recent delivery. The sample was weighted by applying the weighting factor available in the dataset.

### Outcome variable

The outcome variable was the facility for delivery. This was measured from responses to the place of delivery. The responses were grouped into two parts. The first is public health facilities, which consist of deliveries in government hospitals, government health centers, government health posts, and other public sector facilities. This group was coded ‘1’ and represents the category of interest in the study. Two, are private health facilities, which consists of deliveries in private hospital/clinics, other private medical facilities, and other non-public facilities. This category was coded ‘0’.

### Explanatory variables

The main explanatory variable was the antenatal care facility. This was divided into two groups, namely, public health facilities and private health facilities. Included in public health facilities are the antenatal care received at government hospitals, government health centers, government health posts, and other public sector health facilities. Antenatal care received at private hospitals/clinics, and other private medical facilities were included in the non-public health facilities. Based on findings in existing studies important covariates of facility delivery were selected and grouped as predisposing, enabling, and need factors in line with the Andersen model. The predisposing factors examined were maternal age (15–24, 25–34, or 35–49 years), age at first birth (15–19, 20–24, or 25 years or older), parity (primiparity – 1 child, multiparity – 2 to 4 children, or grand multiparity – 5 or more children), exposure to mass media (low, moderate, or high), and, religion (Christianity, Islam, or Others). These variables have been found to be important drivers of facility delivery in existing studies [2, 52–55].

The selected enabling factors were household wealth (poorest, poorer, middle, richer, or richest), work status (employed or unemployed), partners’ education (none, primary, secondary, or higher), women’s autonomy (low or high) based on involvement in household decision-making, health insurance (enrolled or not enrolled), and, perception of distance to the health facility, which may prevent a woman from accessing healthcare. Women’s autonomy was generated from responses to who had the final say in their own healthcare, purchase of large household items, and visits to friends/relatives by creating a composite index. This index has a total score of 12 points with 1–5 points indicating poor autonomy, and 6 points or higher indicating high autonomy. A number of recent studies [37, 29, 56, 57, 3, 58] have established that these variables are significant predictors of facility delivery. Three variables were selected as the need factors in the study. These are pregnancy wantedness (planned or unplanned), the number of antenatal care visits (3 or less, 4–7, or 8 +), and the timing of first antenatal care (first trimester, second trimester, or third trimester). These variables have been well-connected to the uptake of facility delivery in existing studies [3, 29, 52, 54]. In line with the construct of the Andersen model, two, variables were used as measures of the external environment. These are the place of residence (urban or rural), and geographic region (northern or southern). These variables have been used for similar analytical purposes in previous studies [1, 57, 59].

### Data analysis

Statistical analyses were performed with the aid of Stata version 14 [60]. Data were checked for non-response and missing values prior to analysis to ensure that all the variables have the same total frequency. The levels of antenatal and delivery care in public health facilities together with respondents’ profiles were presented using frequency distribution and percentages. The cross-tabulation of the research variables was carried out and presented alongside the frequency distributions. The relationship between the outcome and explanatory variables was examined using the unadjusted Odds Ratio (uOR) with a 95% confidence interval. This was done for the purpose of selecting variables for the multivariable model. Statistical significance at this level was set at p < .025. In addition, a Variance Inflation Factor (VIF) was performed to identify and eliminate collinear variables based on the VIF value. Any variable showing a VIF value of 10 or above was excluded so that the estimation of the regression coefficient is not misleading. This practice is widely applied in regression analysis [61].

At the multivariable level, two binary logistic regression models were fitted. Model 1 was based on antenatal care facilities and the predisposing enabling, and need factors. The essence of this model was to examine whether the inclusion of the additional variables will weaken or strengthen the influence of antenatal facilities on delivery facilities as shown in the unadjusted model. Model 2 was the full model which included all the research variables. Models 1 and 2 estimated the regression coefficients using the adjusted Odds Ratio (aOR) with a 95% confidence interval. Statistical significance was set at the 5% level (p < .05).

## Results

### Univariate and bivariate results

Table 1 presents respondents’ socio-demographic, health characteristic, and level of usage of public and private health facilities for deliveries. Slightly more than two-thirds (67.6%) of the respondents utilized public health facilities for deliveries. Likewise, more than two-thirds (69.6%) of the respondents received antenatal care in public health facilities. Out of this proportion, the majority (91.6%) subsequently had deliveries in public health facilities. However, less than one-tenth (8.4%) of the women who received antenatal care in public health facilities had deliveries in private health facilities. Though women in the 25–34 age group were dominant in the sample (50.8%), the utilization of public health facilities for deliveries was highest among younger women. Nearly two-thirds of the women were less than 25 years at the time of their first birth but the utilization of public health facilities for deliveries declined consistently as age at first birth increased. Primiparous women were dominant in the sample (42.9%). However, the use of public health facilities for deliveries was highest among grand multiparous women (72.4%).

**Table 1:** Usage of public health facilities for deliveries by respondents’ socio-demographic and health characteristics

Characteristic	Frequency (%)	Delivery facility		Characteristic	Frequency (%)	Delivery facility	
		Private (%)	Public (%)			Private (%)	Public (%)
**Antenatal care facility**				**Work status**			
Private facility	2,741 (30.4)	87.3	12.7	Unemployed	2,016 (22.4)	28.9	71.1
Public facility	6,274 (69.6)	8.4	91.6	Employed	6,999 (77.6)	33.4	66.6
**Maternal age**				**Women’s autonomy**			
15–24 years	1,828 (20.3)	24.3	75.7	Low	1,187 (13.1)	44.0	56.0
25–34 years	4,585 (50.8)	33.9	66.1	High	7,829 (86.8)	30.6	69.4
35–49 years	2,602 (28.9)	35.5	64.5	**Partners’ education**			
**Age at first birth**				None	1,683 (18.7)	19.8	80.2
15–19 years	3,384 (37.5)	21.5	78.4	Primary	1,114 (12.3)	30.3	69.7
20–24 years	3,337 (37.0)	33.5	66.5	Secondary	3,987 (44.2)	36.8	63.2
25+	2,294 (25.5)	46.8	53.1	Higher	2,231 (24.7)	35.1	64.9
**Parity**				**Perception of distance to health facility**			
Primiparity	3,872 (42.9)	34.3	65.7	Big problem	1,883 (21.0)	33.7	66.3
Multiparity	2,755 (30.6)	33.9	66.1	Not a problem	7,122 (79.0)	32.1	67.9
Grand multiparity	2,388 (26.5)	27.6	72.4	**Health insurance**			
**Maternal education**				Not enrolled	8,670 (96.2)	32.3	67.7
None	1,474 (16.3)	11.5	88.5	Enrolled	345 (3.8)	35.7	64.3
Primary	1,367 (15.2)	25.7	74.3	**Timing of first antenatal visit**			
Secondary	4,491 (49.8)	37.4	62.6	First trimester	2,682 (29.8)	40.0	60.0
Higher	1,683 (18.7)	43.0	57.0	s trimester	5,356 (59.4)	29.6	70.4
**Mass media exposure**				Third trimester	977 (10.8)	26.6	73.4
Low	1,588 (17.6)	20.0	80.0	**Pregnancy wantedness**			
Moderate	4,417 (49.0)	33.0	67.0	Planned	7,499 (83.2)	31.4	68.6
High	3,010 (33.4)	38.0	62.0	Unplanned	1,516 (16.8)	37.4	62.6
**Religion**				**Number of antenatal visits**			
Christianity	5,486 (60.8)	40.7	59.3	3 or less	1,627 (18.0)	26.8	73.2
Islam	3,495 (38.8)	19.4	80.6	4–7	3,980 (44.2)	25.4	74.6
Others	34 (0.4)	19.7	80.3	8+_	3,408 (37.8)	43.3	56.7
**Household wealth**				**Place of residence**			
Poorest	548 (6.1)	15.4	84.6	Urban	5,412 (60.0)	38.6	61.4
Poorer	1,109 (12.3)	19.8	80.2	Rural	3,604 (40.0)	23.1	76.0
Middle	1,856 (20.6)	25.5	74.5	**Geographic region**			
Richer	2,495 (27.7)	32.0	68.0	Northern	3,808 (42.2)	17.5	82.5
Richest	3,007 (33.4)	44.7	55.3	Southern	5,207 (57.8)	43.3	56.7
**Total**	**9,015 (100.0)**	**32.4**	**67.6**	**Total**	**9,015 (100.0)**	**32.4**	**67.6**

**Source**: Authors’ analyses based on 2018 Nigeria Demographic and Health Survey.

The majority of the women attained some level of education but secondary education was the dominant level attained among the women (49.8%). As maternal education improved from one level to the next higher level, the utilization of public health facilities for deliveries declined consistently among women. Similarly, as mass media exposure improved among women, the utilization of public health facilities for deliveries declined consistently. Christian women were dominant (60.8%) in the sample compared to Muslim women, but Muslim women reported higher usage of public health facilities for deliveries compared to Christian women (80.6% vs. 59.3%). The proportion of women from ‘richer’ or ‘richest’ households was higher among the respondents, but the usage of public health facilities for deliveries was lower among these categories of women.

The majority of the respondents (77.6%) were employed at the time of the survey but the use of public health facilities for deliveries was higher among the unemployed women. In contrast, women who had high autonomy were not only dominant in the sample (86.8%), but they also reported higher utilization of public health facilities for deliveries. Distribution by partners’ education revealed that secondary education was the most common educational level attained by respondents’ partners. Except in the higher education category, as partners’ education improved to the next higher level, the utilization of public health facilities for deliveries declined. The majority of respondents (79.0%) did not perceive the distance to the health facility as a problem. However, this group of women reported a similar usage of public health facilities for deliveries compared to those who perceived distance to the health facility as a big problem. Virtually all the respondents were not enrolled in any health insurance scheme. However, the few women who enrolled in health insurance reported higher usage of private health facilities for deliveries.

The timing of the first antenatal care visit showed that more than half of the respondents (59.4%) initiated their first antenatal care visit in the second trimester of the pregnancy. On the other hand, usage of public health facilities for deliveries revealed that utilization of public health facilities for deliveries was highest among women who initiated antenatal care visits in the third trimester of pregnancy. The majority of the respondents (83.2%) planned their most recent delivery and reported higher usage of public health facilities for deliveries. More women (44.2%) had between three to seven antenatal care attendance, and higher usage of public health facilities for deliveries was also observed among them. Urban women were dominant in the sample (60.0%) but rural women reported higher usage of public health facilities for deliveries. Similarly, southern women were dominant among the respondents (57.8%) but northern women reported higher utilization of public health facilities for deliveries. Table 2 presents the relationships between the research variables. Based on the uOR, two variables, namely, perception of distance to a health facility, and health insurance enrolment were not significantly related to deliveries in public health facilities. These variables were thus, excluded from further analysis.

**Table 2:** Relationship between delivery in public health facilities and socio-demographic and health characteristics

Characteristic	uOR	p > |t|	95% CI	Characteristic	uOR	p > |t|	95% CI
**Antenatal care facility**				**Work status**			
Private health facility	-	-	-	Unemployed	-	-	-
Public health facility	5.12***	p < .01	4.39–5.97	Employed	0.81***	p < .01	0.71–0.93
**Maternal age**				**Women’s autonomy**			
15–24 years	-	-	-	Low	-	-	-
25–34 years	0.63***	p < .01	0.53–0.74	High	1.78***	p < .01	1.48–2.14
35–49 years	0.58***	p < .01	0.49–0.69	**Partner’s education**			
**Age at first birth**				None	-	-	-
15–19 years	-	-	-	Primary	0.57***	p < .01	0.45–0.71
20–24 years	0.54***	p < .01	0.47–0.63	Secondary	0.42***	p < .01	0.35–0.51
25 + years	0.31***	p < .01	0.26–0.37	Higher	0.46***	p < .01	0.38–0.55
**Parity**				**Perception of distance to health facility**			
Primiparity	-	-	-	Big problem	-	-	-
Multiparity	1.02	0.81	0.89–1.15	Not a problem	1.08	0.39	0.91–1.27
Grand multiparity	1.37***	p < .01	1.18–1.59	**Health insurance**			
**Maternal education**				Not enrolled	-	-	-
None	-	-	-	Enrolled	0.86	0.33	0.63–1.17
Primary	0.37***	p < .01	0.29–0.48	**Timing of first antenatal visit**			
Secondary	0.22***	p < .01	0.17–0.27	First trimester	-	-	-
Higher	0.17***	p < .01	0.13–0.22	s trimester	1.58***	p < .01	1.40–1.79
**Mass media low exposure**				Third trimester	1.84***	p < .01	1.50–2.26
Low	-	-	-	**Pregnancy wantedness**			
Moderate	0.51***	p < .01	0.42–0.61	Planned	-	-	-
High	0.41***	p < .01	0.32–0.52	Unplanned	0.76***	p < .01	0.66–0.88
**Religion**				**Number of antenatal visits**			
Christianity	-	-	-	3 or less	-	-	-
Islam	2.85***	p < .01	2.38–3.42	4–7	1.07	0.40	0.91–1.27
Others	2.81***	p < .01	1.17–6.74	8+	0.48***	p < .01	0.40–057
**Household wealth**				**Place of residence**			
Poorest	-	-	-	Urban	-	-	-
Poorer	0.74**	0.04	0.55–0.99	Rural	2.10***	p < .01	1.72–2.55
Middle	0.53***	p < .01	0.39–0.72	**Geographic region**			
Richer	0.39***	p < .01	0.28–0.53	Northern	-	-	-
Richest	0.22***	p < .01	0.16–0.31	Southern	0.28***	p < .01	0.23–0.34

### Multivariable results

Table 3 presents the effects of the socio-demographic and health characteristics on the utilization of public health facilities for deliveries. The inclusion of the predisposing, enabling, and need factors in Model 1 did not undermine the influence of antenatal care facilities on public health facilities. Women who received antenatal care in public health facilities were more than six times more likely to utilize public health facilities for deliveries (aOR = 6.38, 95% CI: 5.32–7.65). In the model, maternal age, and mass media exposure were the two variables that failed to exert a significant influence on the likelihood of deliveries in public health facilities. The inclusion of the environmental context in Model 2 did not result in any substantial change in the effects on the utilization of public health facilities for deliveries. In the model, four predisposing factors (age at first birth, parity, maternal education, and religion), four enabling factors (household wealth, work status, women’s autonomy, and partners’ education), and three need factors (the timing of first antenatal care contact, pregnancy wantedness, and the number of antenatal care visits) were significant predictors of the odds of utilizing public health facilities for deliveries.

**Table 3:** Effects of socio-demographic and health characteristics on usage of public health facilities for deliveries

Characteristic predicting delivery in public health facilities	Model 1			Model 2		
	**aOR**	**p > |t|**	**95% CI**	aOR	**p > |t|**	95% CI
**Antenatal care facility**						
Private health facility	1.00	-	-	1.00	-	-
Public health facility	6.38***	p < .01	5.32–7.65	6.82***	p < .01	5.52–8.42
**Maternal age**						
15–24 years	1.00	-	-	1.00	-	-
25–34 years	1.03	0.87	0.73–1.45	1.09	0.61	0.78–1.53
35–49 years	0.99	0.99	0.63–1.59	1.07	0.77	0.68–1.69
**Age at first birth**						
15–19 years	1.00	-	-	1.00	-	-
20–24 years	0.74**	0.01	0.59–0.92	0.78**	0.03	0.63–0.98
25 + years	0.52***	p < .01	0.38–0.71	0.56***	p < .01	0.41–0.76
**Parity**						
Primiparity	1.00	-	-	1.00	-	-
Multiparity	0.90	0.43	0.70–1.16	0.89	0.37	0.69–1.14
Grand multiparity	0.81***	p < .01	0.73–0.89	0.52***	p < .01	0.47–0.58
**Maternal education**						
None	1.00	-	-	1.00	-	-
Primary	0.52***	p < .01	0.36–0.74	0.56***	p < .01	0.39–0.80
Secondary	0.38***	p < .01	0.26–0.55	0.43***	p < .01	0.29–0.62
Higher	0.36***	p < .01	0.23–0.57	0.40***	p < .01	0.25–0.62
**Mass media low exposure**						
Low	1.00	-	-	1.00	-	-
Moderate	0.84	0.18	0.64–1.09	0.92	0.54	0.71–1.20
High	1.12	0.45	0.84–1.49	1.27	0.10	0.95–1.71
**Religion**						
Christianity	1.00	-	-	1.00	-	-
Islam	1.61***	p < .01	1.25–2.07	1.40**	0.01	1.08–1.82
Others	1.78	0.25	0.67–4.74	2.02	0.18	0.72–5.67
**Household wealth**						
Poorest	1.00	-	-	1.00	-	-
Poorer	0.92	0.68	0.61–1.38	0.94	0.78	0.63–1.41
Middle	0.78	0.29	0.49–1.23	0.89	0.63	0.56–1.41
Richer	0.78	0.29	0.49–1.23	1.03	0.88	0.65–1.63
Richest	0.57**	0.02	0.35–0.92	0.62***	p < .01	0.58–0.69
**Work status**						
Unemployed	1.00	-	-	1.00	-	-
Employed	1.74***	p < .01	1.51–2.01	1.30***	p < .01	1.16–1.48
**Women’s autonomy**						
Low	1.00	-	-	1.00	-	-
High	1.13**	0.02	1.02–1.25	1.87***	p < .01	1.62–2.17
**Partner’s education**						
None	1.00	-	-	1.00	-	-
Primary	0.68**	0.02	0.50–0.95	0.74	0.06	0.53–1.01
Secondary	0.79	0.10	0.60–1.04	0.75**	0.04	0.57–0.99
Higher	0.82	0.29	0.57–1.18	0.73	0.09	0.50–1.06
**Timing of first antenatal visit**						
First trimester	1.00	-	-	1.00	-	-
Second trimester	0.98	0.82	0.79–1.20	1.01	0.88	0.82–1.26
Third trimester	4.48***	p < .01	2.99–6.71	4.50***	p < .01	3.02–6.71
**Pregnancy wantedness**						
Planned	1.00	-	-	1.00	-	-
Unplanned	0.73**	0.01	0.57–0.92	0.75**	0.02	0.59–0.96
**Number of antenatal visits**						
3 or less	1.00	-	-	1.00	-	-
4–7	0.81	0.22	0.59–1.13	0.80	0.18	0.57–1.11
8+	0.60***	p < .01	0.43–0.84	0.70**	0.04	0.50–0.98
**Place of residence**						
Urban				1.00	-	-
Rural				1.41**	0.01	1.11–1.79
**Geographic region**						
Northern				1.00	-	-
Southern				0.55***	p < .01	0.42–0.71

**Notes**: RC (Reference category), **p < .05, ***p < .01

Furthermore, in Model 2, women who received antenatal care in public health facilities were more than six times more likely to use public health facilities for deliveries compared to other women (aOR = 6.82; 95% CI: 5.52–8.42). Older age at first birth was associated with lower odds of utilizing public health facilities for deliveries. Likewise, grand multiparous women were less likely to use public health facilities for deliveries compared to primiparous women (aOR = 0.52, 95% CI: 0.47–0.58). The likelihood of utilizing public health facilities decreased with improvement in the educational level attained. Muslim women were more likely to utilize public health facilities for deliveries compared to Christian women (aOR = 1.40; 95% CI: 1.08–1.82). The likelihood of utilizing public health facilities for deliveries reduces only when household wealth improves to the next richest level (aOR = 0.62, 95% CI: 0.58–0.69). Employed women were more likely to utilize public health facilities for deliveries compared to unemployed women (aOR = 1.30; 95% CI: 1.16–1.48). The likelihood of utilizing public health facilities for deliveries was higher among women who had a high level of autonomy compared to women who had low autonomy (aOR = 1.87, 95% CI: 1.62–2.17). The odds of utilizing public health facilities for deliveries reduce as women’s partners attained improved education. While women who initiated antenatal contact in the third trimester of pregnancy had higher odds of utilizing public health facilities for deliveries (aOR = 4.50, 95% CI: 3.02–6.71), the odds were lower among women who had unplanned pregnancies (aOR = 0.75, 95% CI: 0.59–0.96), and women who had eight or more antenatal attendance (aOR = 0.70; 95% CI: 0.50–0.98). Rural women were more likely to have public health facility delivery compared to urban women (aOR = 1.41; 95% CI: 1.11–1.79), while southern women had lower odds of public health facility delivery compared to northern women (aOR = 0.55; 95% CI: 0.42–0.71).

## Discussion

This study reports that antenatal care in public health facilities is significantly associated with deliveries in public health facilities in Nigeria. Findings reveal that Nigerian women tend to have child deliveries in the sector where antenatal care was received. As evident in the study, the majority of women who received antenatal care in public health facilities had deliveries in public health facilities, and the majority of those who received antenatal care in private health facilities equally had deliveries in private health facilities. This finding may be reasoned in two ways. On one hand, the finding suggests that in spite of the challenges associated with the use of public health facilities in the country [32, 35, 38], many women may prefer to remain within the sector to access other needed maternal healthcare services possibly because the public sector facilities may be the affordable maternal healthcare services to women from all socio-economic backgrounds [16–18]. This is an indication that governments at all levels in the country should ensure expanded availability of public health facilities in all parts of the country. On the other hand, women who patronized private health facilities are most likely to be those who are able to afford the high cost of private health care, and in the absence of serious complications, such women are not likely to switch services to the public sector. Regardless of the sector of antenatal or delivery care, it is important to devise more measures that strengthen better connections between one level of care to another level of care within and between the different sectors of the maternity care delivery system in the country. This will promote women’s health and safe motherhood through adequate access to needed maternal healthcare services. Three other key implications emerged from the findings.

One, the higher patronage of public health facilities for maternity may continue to have serious implications for public health financing, which is rarely sufficient to address the growing health needs of the population. In many parts of the country, a user fee removal policy is being implemented in respect of maternal and child health services [62–64], though some unofficial costs might still be incurred in the process of utilizing these services [65], virtually all the cost-of-service provision is incurred by the government. This high cost needs to be reduced due to two reasons. Firstly, sustained high public spending on maternity care delivery may delay the government’s capacity to expand the available health infrastructure, particularly in the Northern and rural parts of the country where the utilization of facility delivery is the poorest in the country. Secondly, there is a need to scale up the utilization of private health facilities for both antenatal and delivery care. Many childbearing women may not be patronizing private hospitals/clinics for maternity care in many developing countries due to the higher cost of services compared to the public sector facilities [66]. In private health facilities, women even in rural areas are expected to pay for appointments with obstetricians, drugs, tests and ultrasound scans, and antenatal classes [67]. This increases the costs of services and made many women and their partners erroneously believe that private health facilities are driven by profit motives.

Two, the ultimate effect of low patronage of private health facilities for maternity care is sub-optimal use of the private sector in improving facility delivery across developing countries [68]. Private health facilities need to be assisted to effectively play their role as a substitute for public health facilities. Though, promoting the private sector facility without corresponding improvement of the public sector facility may widen the disparity between poor and rich women in terms of access to healthcare, which is not good for the attainment of health equity in the country. Notwithstanding, two actions by the government may make private health facilities affordable to more women who desire the services. On one hand, the federal government of Nigeria, as well as the health development partners should devise strategies to reduce the cost of operating private health facilities in the country. For example, the government can provide tax rebates, and subsidies on the importation of medical equipment to reduce the cost of operating formal private health facilities in the country. Though in the short run, granting tax relief to private health facilities may reduce government revenue, in the long run, public spending on health may reduce once the private sector becomes a key player in health delivery in the country. However, these strategies required the implementation of an effective regulatory/supervisory framework [69], which is important for the provision of quality services. On the other hand, the government could step up efforts to encourage more women to enroll in the available health insurance schemes in the country. As revealed in the study, enrolment for health insurance schemes is very poor in the country and needs to be scaled up to help more women afford the cost of private healthcare services.

Three, the study revealed sets of predisposing, enabling, and need factors that significantly associate with deliveries in public health facilities. In agreement with findings in existing studies, it was found that age at first birth [55], grand multiparity [3, 37, 53], maternal education [54], religion [54], and the timing of the first antenatal visit [54], and numbers of antenatal care visits [52, 59] were important correlates of the utilization of facility delivery. It is important that these variables remain critical targets of preconception health programs through the effective mobilization of both young and older women of childbearing age in the community. A lot could be achieved by designing additional public health educational programs that exemplify how these variables adversely affect service utilization. The behavior change communication strategy of the 2019 national health promotion policy [46] could be expanded to accommodate the implications of these variables. This strategy should also include providing more public health education on the dangers of sub-optimal antenatal care visits and delaying the first antenatal care visits by pregnant women in the country. Also, findings showed that household wealth, working status, women’s autonomy, and partner education were significantly associated with the use of public health facilities for delivery. This not only agrees with findings in previous studies [29, 56, 57], but it also suggests that improving the social conditions of men and women, particularly in rural areas through social development programs may boost the uptake of facility delivery in many developing countries.

### Strengths and limitations

The study filled an important knowledge gap by examining how well antenatal care in public health facilities was associated with deliveries in public health facilities, which was largely ignored in many existing studies that linked antenatal and delivery care. The findings in the study provide more support for the application of the Andersen behavioral model of health services use. The study thus made contributions to the existing body of knowledge in respect of facility delivery. The nationally representative data analyzed in the study are available in the public domain, and with the description of the study variables, findings in the study could be confirmed by other researchers. Notwithstanding, the use of cross-sectional data in the study may not permit establishing actual causality between antenatal and delivery care in public health facilities. It is however worthy of note that the association between antenatal and delivery care in public health facilities as found in the study is strong enough to imply that antenatal care in a public health facility is a strong driver of public health facility delivery. Also, findings in the study suggest the possibility of some women who did not receive ANC in public health facilities do have deliveries in public health facilities. However, the data analyzed did not capture possible reasons for this health behavior. We suggest a follow-up study using the qualitative procedure to shed more light on this health behavior. In Addition, the analysis was based on self-report, which may be affected by recall bias during the survey.

## Conclusion

Based on further analysis of the Nigeria demographic and health survey data, the study provides additional evidence that public health facilities are not only more patronized for antenatal care, but also that patronage is significantly associated with delivery in public health facilities in the country. This finding implies that many childbearing women continue to utilize public health facilities in spite of existing challenges because they may be affordable facilities for women of all socio-economic backgrounds. It is imperative for governments in the country to take more steps to ensure the expanded availability of public health facilities in all parts of the country since their use for antenatal care is well-associated with their use for delivery care. Also, it is important that more measures are devised to strengthen the connection between antenatal and delivery care within the public sector of the maternity delivery system in the country. There is equally a need to reduce the cost of accessing maternity care in private health facilities to make the sector affordable to more women who might need the services.

## Data Availability

Data analyzed could be accessed online at. https://dhsprogram.com/data/dataset/Nigeria_Standard-DHS_2018.cfm?flag=1.

## List of abbreviations

DHS: Demographic and Health Survey
FMoH: Federal Ministry of Health
NDHS: Nigeria Demographic and Health Survey
NPC: National Population Commission
